# Supplementary material for: The interactome of intact mitochondria by cross-linking mass spectrometry provides evidence for coexisting respiratory supercomplexes
Source: Mol Cell Proteomics. 2017 Dec 8;17(2):216–32. doi: 10.1074/mcp.RA117.000470 (PMC5795388; doi:10.1074/mcp.RA117.000470)
Supplement: Supplemental Data [file supp_RA117.000470_133922_0_supp_23978_fzffwf.zip › spectra_annotation/mito_DR_spectra_annotation/454-1-7-1-12-1.pdf]

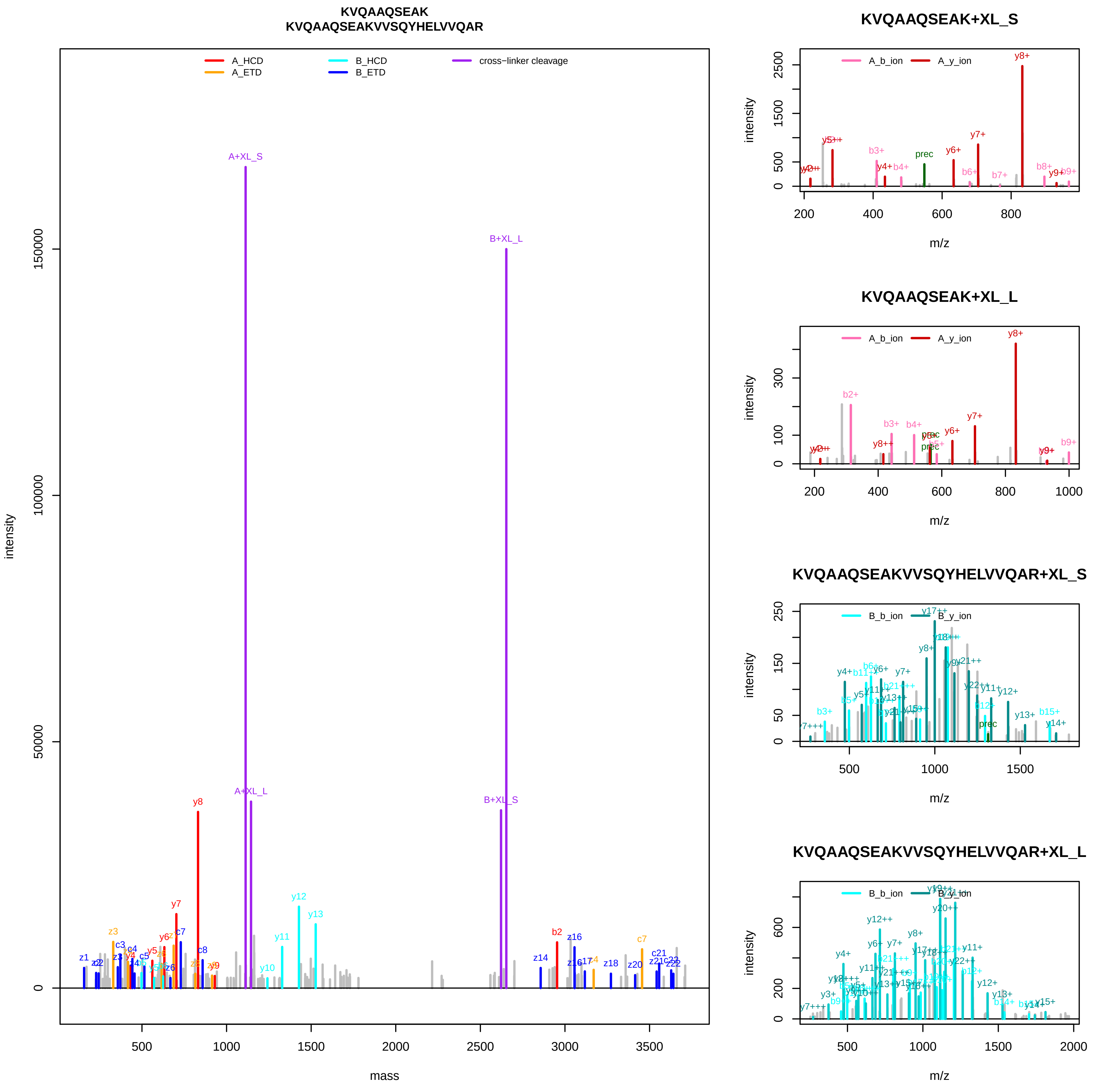
KVQAAQSEAK
KVQAAQSEAKVVSQYHELVVQAR
A_HCD
A_ETD
B_HCD
B_ETD
cross−linker cleavage
A+XL_S
B+XL_L
A+XL_L
B+XL_S
y8
y7
y12
y13
y11
y10
b2
z16
z14
z18
z20
c7
c8
c21
z3
c3
c4
c5
y6
y5
y4
y9
z1
z2
z6
intensity
mass
0
50000
100000
150000
500
1000
1500
2000
2500
3000
3500
KVQAAQSEAK+XL_S
A_b_ion
A_y_ion
y8+
y7+
y6+
prec
b3+
y4+
b4+
b6+
b7+
b8+
y9+
b9+
intensity
m/z
KVQAAQSEAK+XL_L
b2+
b3+
b4+
y8++
b5+
KVQAAQSEAKVVSQYHELVVQAR+XL_S
B_b_ion
B_y_ion
y17++
y8+
y4+
y12+
y13+
b15+
y14+
b3+
KVQAAQSEAKVVSQYHELVVQAR+XL_L
y20++
y12++
y11+
y15+
